# Effect of modified subcostal anterior quadratus lumborum block on perioperative opioid consumption in patients undergoing gynecologic endoscopic surgery

**DOI:** 10.3389/fonc.2025.1539241

**Published:** 2025-05-19

**Authors:** Chunchao Peng, Guohui Fu, Jingping Chai, Jide A, Wenhui Guang

**Affiliations:** ^1^ Department of Anesthesiology, Qinghai Provincial People’s Hospital, Xining, China; ^2^ Department of Internal Medicine-Cardiovascular, Qinghai Provincial People’s Hospital, Xining, China; ^3^ Department of General Surgery, Qinghai Provincial People’s Hospital, Xining, China

**Keywords:** ultrasonic guidance, anterior quadratus lumborum block, gynaecologicendoscopic surgery, opioid use, multimodal analgesia

## Abstract

**Objective:**

To assess the impact of ultrasound-guided multimodal anesthesia utilizing a modified subcostal anterior quadratus lumborum block (QLB) in conjunction with general anesthesia on perioperative opioid consumption among patients undergoing gynecologic endoscopic surgery.

**Methods:**

A total of 56 patients aged 18–65 years, classified as ASA physical status I-II, with a BMI of 18–30 kg.m², were recruited from Qinghai Provincial People’s Hospital between June 2023 and August 2024 for elective laparoscopic gynecological surgery. According to the random number table method, patients were randomly allocated into two groups: the improved subcostal border anterior quadrate block combined with general anesthesia group (Group A) and the traditional anterior quadrate block combined with general anesthesia group (Group B), each comprising 28 patients. Both groups underwent tracheal intubation and general anesthesia. Before anesthesia induction, patients in Group A received 0.33% ropivacaine (20 ml per side) administered bilaterally under ultrasound guidance for the improved anterior quadratus lumborum block. In contrast, patient in Group B received 0.33% ropivacaine (20 ml per side) administered bilaterally under ultrasound. Following the surgical procedure, both groups were administered controlled patient-controlled intravenous analgesia (PCIA). The administration of perioperative opioids (including intraoperative remifentanil dosage and postoperative opioid dosage) as well as propofol was systematically recorded during the follow-up period; VAS scores were recorded both at dynamic and static at 2 hours, 6 hours,–24 hours, and 48 hours post-intervention; The number of effective analgesic pump activations within–48 hours post-operation was recorded. Additionally, the time of the first anal exhaust and the time of feeding within 48 hours after surgery were documented. Postoperative adverse reactions, including skin itching, nausea or vomiting, and dizziness, were also observed.

**Results:**

Compared to Group B, Group A exhibited a significantly lower dosage of remifentanil (1.49 ± 0.50 mg vs 1.86 ± 0.77 mg, P<0.05) and postoperative opioids (median, 31.79 μg with IQR 23.04-42.75μg vs median, 42.30μgwith IQR 43.26-44.64μg), P<0.05); The dynamic and static VAS scores of patients in Group A were significantly reduced at 2 hours (median 3.00 with IQR 2.00-3.00 vs median 4.00 2with IQR 4.00-4.50, median 3.00 with IQR 2.00-3.00 vs median 3.00 with IQR 3.00-4.00, P<0.001), 6 hours (median 3.00 with IQR 2.00-3.50 vs median 4.00 with IQR 4.00-4.50, median 2.00 with IQR 2.00-3.00 vs median 3.50 with IQR 3.00-4.00, P<0.001) and 24 hours (median 3.00 with IQR 3.00-4.00 vs median 4.00 with IQR 3.50, 4.50, median 3.00 with IQR2.00-3.00 vs median 3.00 with IQR 3.00-4.00, P<0.05); There was no statistically significant difference in dynamic and static VAS scores between the two groups at 48 hours (P=0.568, P = 0.109); The number of analgesic pump compressions in Group A significantly decreased at 48 hours post-surgery (median,0.00 with IQR 1.00-2.00) vs median,1.50 with IQR 0.25-4.00, P<0.05). There was no statistically significant difference in the propofol dosage between the two groups (P=0.667); The A group achieved earlier oral feeding (median 25.00 h with IQR 20.00-30.00 h vs median 33.25 h with IQR 21.50-38.00 h,P<0.05), earlier anal release of gas (median 24.00 h with IQR 21.00-30.00 h vs median 32.00 h with IQR 24.50-38.00, P<0.05); Compared with Group B, the incidence of postoperative dizziness (10% vs 21%, P<0.05), nausea or vomiting (4% vs 17%, P<0.001), and skin pruritus(0% vs 9%, P<0.05) in Group A was significantly reduced (P<0.05).

**Conclusion:**

Compared to the traditional anterior quadratus lumborum block, the modified subcostal edge anterior quadratus lumborum block significantly decreases perioperative opioid consumption in gynecological laparoscopic surgery patients, effectively alleviates postoperative pain, accelerates gastrointestinal function recovery, and minimizes adverse reactions.

## 1 Introduction

Laparoscopic gynecological surgery is a commonly employed method for treating benign gynecological conditions, and general anesthesia is typically administered during the procedure to ensure its successful execution (1). Although this surgical method has the advantages of less intestinal interference and less tissue damage (2), the surgical procedure and pneumoperitoneum stimulation may cause trauma, thereby eliciting a stress response and leading to significant postoperative somatic and visceral pain. Patients frequently experience fear of pain, which can result in avoidance of coughing or mobilization, thus increasing the risk of complications such as atelectasis, lung infections, and limb dysfunction. Additionally, varying levels of postoperative pain may necessitate an increased dosage of opioid analgesics. However, Opioids may lead to postoperative gastrointestinal and respiratory adverse reactions (3), thereby impacting the process of postoperative recovery. In recent years, as nerve block technology has continued to advance, a range of nerve block techniques have been validated as effective methods for reducing postoperative pain and decreasing opioid consumption. Compared to fascia block techniques in other abdominal and lumbar regions, the quadratus lumborum block can selectively target the thoracolumbar nerves by modifying the puncture approach. This is achieved through precise adjustment of the puncture depth and direction based on the analgesic requirements of different anatomical regions, thereby effectively accomplishing nerve blockade (4). Studies have demonstrated (5) that the anterior quadratus lumborum block exhibits a significant analgesic effect in abdominal surgery and represents one of the regional nerve block techniques. Drugs administered around the quadratus lumborum muscle can diffuse extensively, alleviating both incisional and visceral pain while reducing perioperative opioid consumption (6). However, as a deep blockade technique, the anterior approach to the quadratus lumborum block can provide extensive analgesia but is also associated with certain risks. It has been reported that this approach may lead to a more pronounced motor blockade, thereby prompting the development of the subcostal transverse abdominis plane block as an alternative. The subcostal anterior quadratus lumborum block is an innovative truncal nerve block technique that builds upon the principles of the anterior quadratus lumborum block, with the block site ascending to the subcostal T12 level (7). Elsharkawy et al. confirmed through cadaveric studies that injecting pigment anterior to the quadratus lumborum muscle at the subcostal level allows the spread of the injectate from the lumbar region to the thoracic paravertebral space. This occurs via the space beneath the arcuate ligament of the lateral diaphragm, forming lacunar spaces deep within the transverse fascia and intrathoracic fascia (8). This modification of the traditional anterior quadrate block leverages the pathway between the lumbar and thoracic paravertebral spaces to facilitate more cephalad spread of the injection by positioning it as close to the arcuate ligament as possible (7), with the blockade range extending from T6 to L4. Other studies have demonstrated that the subcostal and anterior quadratus lumborum block exhibits advantages over the traditional anterior quadratus lumborum block, including a higher blockade plane, prolonged duration of action, and reduced incidence of adverse reactions. Additionally, it holds potential for visceral pain blockade (9). However, in the traditional subcostal anterior QLB, the presence of the transverse fascia and double-layer retrorenal fascia on the ventral side of the quadratus lumborum makes it difficult to distinguish the fascia layer, which can affect the success rate of the block. Therefore, we refined the conventional subcostal and anterior quadrate block technique. At the T12 level, the patient was positioned supine, and under ultrasound guidance, 20 ml of 0.33% ropivacaine was administered between the psoas major and quadratus lumborum muscles, near the spine, using a puncture needle directed from the abdominal side toward the dorsal side. The aim of this study was to assess the impact of a modified subcostal and anterior quadratus lumborum block on perioperative opioid consumption in patients undergoing gynecologic endoscopic surgery, and to compare its efficacy with that of the traditional anterior quadratus lumborum block.

## 2 Materials and methods

### 2.1 General information

A total of 60 patients aged 18–65 years, classified as ASA Physical Status I-II with a BMI of 18–30 kg.m², who underwent elective laparoscopic gynecological surgery (hysterectomy) between June –2023 and August 2024, were included in the study. Based on the exclusion criteria: Severe dysfunction of the heart, lungs, liver, or kidneys, contraindications for the quadratus lumborum block syndrome, cognitive or communication impairments, a history of low back or abdominal surgery, hypersensitivity to local anesthetics, scoliosis, hematological or coagulation disorders, a history of neuromuscular or psychiatric conditions, and long-term use of sedatives or analgesics; Exclusion criteria: Patients with severe abdominal adhesions who underwent laparotomy and had their postoperative analgesia regimen modified were excluded. Finally, 56 patients were randomly allocated into two groups: the improved subcostal border anterior quadrate block combined with general anesthesia group (Group A, n = 28) and the traditional anterior quadrate block combined with general anesthesia group (Group B, n = 28). This study received approval from the Ethics Committee (approval number: (2023)-085), and written informed consent was obtained from the patients or their families prior to surgery.

### 2.2 Randomized blind method

We randomly allocated patients into two groups using a computer-generated list of random numbers via SPSS 25.0 (IBM, Chicago, IL, USA). Sealed opaque envelopes were prepared for each patient by an anesthesia assistant (C1) who was blinded to the grouping assignment and study design. On the morning of surgery, the envelopes were opened by the anesthesiologist (C2), who then prepared and administered the nerve block according to the group assignment. Anesthesia induction and management were performed by the chief anesthesiologist (C3), who remained unaware of the trial design and grouping. Post-operative data were collected by another anesthesiologist (C4), who was also blinded to the grouping. Both the surgeons, statistical analysts, patients were kept blinded throughout the study. All patients were informed of their group allocation upon discharge, and the researchers in this group were unblinded only after the completion of statistical analysis.

### 2.3 Method of anesthesia

The patients fasted for 8 hours and abstained from fluid intake for 4 hours prior to the operation, and did not receive any preoperative medication. Upon entering the operating room, routine oxygen administration was initiated, and venous access was established. Standard monitoring of ECG, heart rate, blood pressure, oxygen saturation, body temperature, PETCO_2_, and BIS values was performed. Two groups of patients received anesthesia induction with midazolam 0.02 mg/kg, sufentanil 0.5 μg/kg, etomidate 0.2 mg/kg, and rocuronium 0.6 mg/kg. Following induction, mask ventilation was performed for two minutes, followed by tracheal intubation and mechanical ventilation with a tidal volume (VT) of 6–8 ml/kg and a respiratory rate (RR) of 11–15 breaths per minute. End-tidal carbon dioxide (PETCO2) was maintained at 35–45 mmHg throughout the procedure. Anesthesia was maintained using a combination of propofol (4–12 mg/kg/h) delivered via intravenous pump, remifentanil (0.05-0.3 μg/kg/min), and intermittent intravenous boluses of rocuronium to ensure muscle relaxation. Bispectral Index (BIS) values were kept within the range of 40–60 throughout the procedure. Before the induction of anesthesia, the patient was positioned in a supine position, with an integrated positional pad placed under the waist if necessary. Scanning was performed using the low-frequency convex array probe (2 ~ 5 MHz) of the ultrasound device. In Group A, the anatomic location of the quadratus lumborum muscle was fully exposed. The ultrasound probe was positioned at the T12 level for a short-axis scan, with the probe marker oriented ventrally to accurately identify the positions of the kidney, quadratus lumborum, psoas major, and spine. The final probe placement position was then marked. Position the ultrasound probe at the pre-marked scanning location and make fine adjustments to the positioning as needed. Perform an in-plane needle insertion, advancing the needle from the ventral side toward the dorsal side. Once the needle tip is confirmed to be positioned between the quadratus lumborum and psoas major muscles, administer 3 ml of normal saline. After verifying adequate spread of the liquid, inject 20 ml of 0.33% ropivacaine. Repeat the same procedure on the contralateral side. The ultrasound probe in Group B was positioned between the iliac crest and the costal margin, subsequently moved to the level of the midaxillary line to verify the location of the psoas major muscle and quadratus lumborum. Once the correct position was confirmed, the final probe placement was marked, followed by disinfection and covering with a sterile drape. The ultrasound probe was then enclosed within a sterile sheath. Subsequently, the probe was repositioned at the pre-marked scanning site, with minor local adjustments as needed. Upon guiding the needle tip to the interfascial plane between the quadratus lumborum and psoas major muscles, 3 mL of normal saline was injected for confirmation of spread. Following verification of adequate fluid dispersion, 20 mL of 0.33% ropivacaine was administered. The procedure on the contralateral side was performed using an identical technique. If intraoperative hypertension exceeded 20% of the baseline value and persisted for more than one minute, a 5 μg intravenous dose of sufentanil was administered. If intraoperative hypotension decreases by more than 20% of the baseline value and persists for over 1 minute, 6 mg of ephedrine should initially be administered intravenously. If blood pressure does not increase after 5 minutes, the infusion rate should be appropriately increased. If intraoperative tachycardia occurs (defined as an increase in heart rate exceeding 20% of the baseline value or a rate faster than 120 beats per minute, lasting for more than 1 minute), metoprolol at a dose of 0.05 mg/kg will be administered intravenously over a period of 2 minutes. In the event of intraoperative bradycardia (defined as a decrease in heart rate exceeding 20% of the baseline value or a rate slower than 60 beats per minute for a duration exceeding 1 minute), atropine at a dose of 0.2 mg will be administered intravenously. Postoperatively, patient-controlled intravenous analgesia (PCIA) was administered, and both patients and their families were provided with detailed instructions on the appropriate use of the analgesia pump. Analgesic pump drug formulation: Sufentanil 100 μg, Ondansetron 8 mg, and 0.9% sodium chloride solution (94 mL). The patient-controlled intravenous analgesia (PCIA) dose was set at 2 mL per administration, with a background infusion rate of 1.5 mL/h and a lockout interval of 25 minutes. If the Visual Analog Scale (VAS) pain score at rest remains greater than 4 despite activation of the analgesic pump, supplemental analgesia should be administered (intravenous Dezocine 5 mg).

### 2.4 Observation indicators

Primary outcome measures: postoperative opioid dosage. Secondary observation metric: Propofol dosage; intraoperative remifentanil dosage; VAS scores at rest and during exercise at 2, 6, 24, and 48 hours post-surgery; the number of times the patient pressed the postoperative analgesic pump within–48 hours; the first time of anal exhaust and feeding within 48 hours post-surgery; and the occurrence of postoperative adverse reactions such as skin itching, nausea, vomiting, and dizziness (The clinician evaluated the patient’s subjective symptoms at 2, 6, 24, and 48 hours post-treatment).

### 2.5 Statistical analysis

In this study, postoperative opioid consumption was used as the main observation index, and PASS15 software was utilized. Our preliminary pilot study with 14 patients showed that the postoperative opioid dosage were 22.03 ± 6.14(mean ± SD, group A) and 30.98 ± 9.62(mean ± SD, groupB).Based on this pilot study, we calculated a minimum sample size of 26 patients in each group with a two sided alpha level of 0.05 and a power of 0.9. To account for a 10% dropout rate and follow-up failure,–30 patients were recruited in each group.

Statistical analyses were performed using SPSS 25.0 software. Measurement data were assessed for normality using the Shapiro-Wilk Test (SW). Normally distributed measurement data were presented as mean ± standard deviation (¯x ± s), and intergroup comparisons were conducted using the T-test. Non-normally distributed measurement data were expressed as median M (P25, P75), with intergroup comparisons analyzed via the rank sum test. Categorical data were reported as frequency (%), and comparisons were made using the χ2 test or Fisher’s exact test where appropriate. A p-value of less than 0.05 was considered statistically significant.

## 3 Results

### 3.1 General situation of the two groups

**Figure 1:**
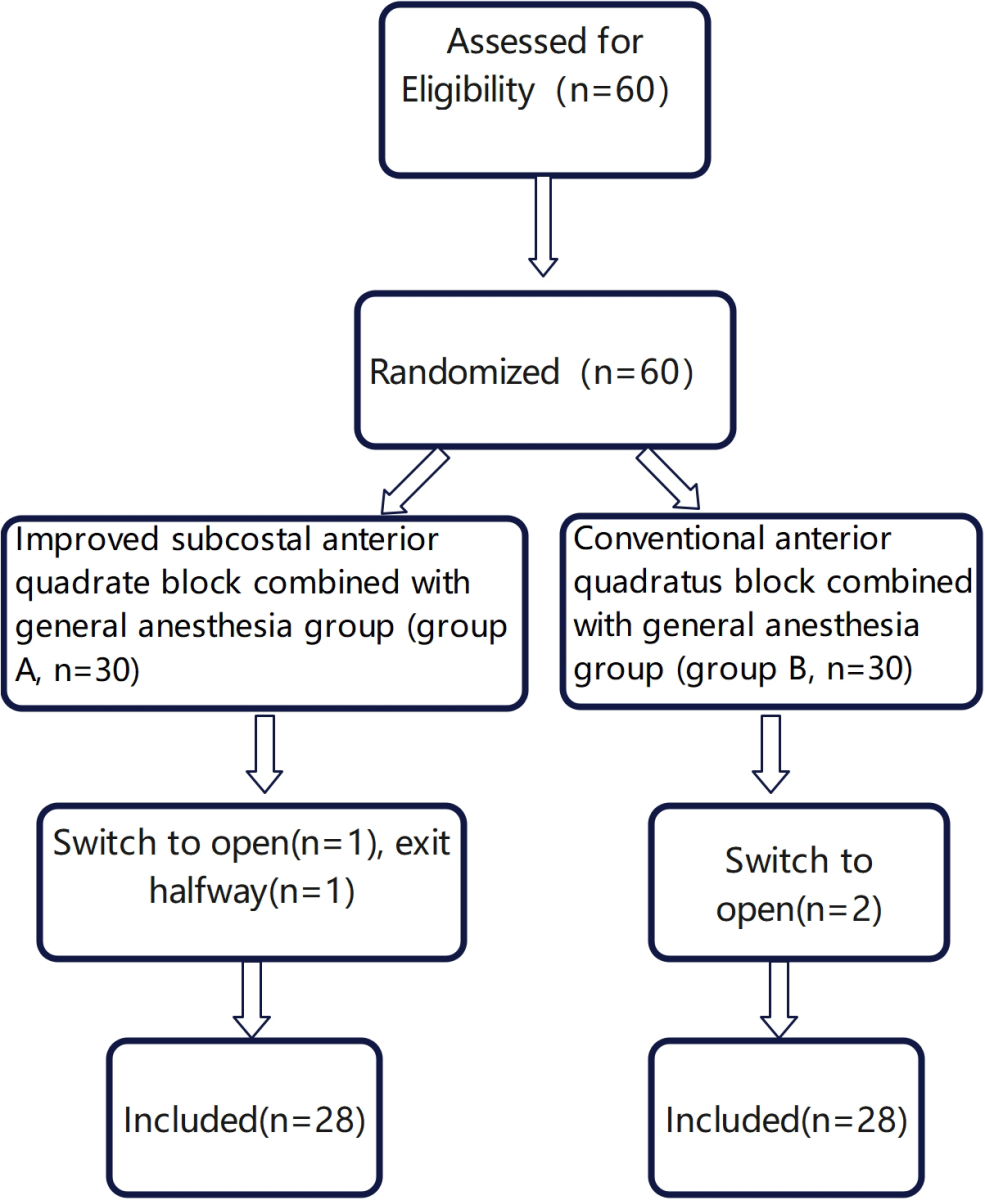
Patient selection flow chart.

In this study, a total of 60 patients were initially enrolled. Among them, three patients underwent conversion to laparotomy during the operation(two due to severe adhesions and one because of lesion invasion into surrounding tissues). Additionally, one patient withdrew from the study post- surgery. Consequently, the final analysis included 56 patients, with 28 patients in each group (**Figure 1**). There were no statistically significant differences in age, height, weight, or operation duration between the two groups (P≥0.05) (**Table 1**).

**Table 1:** Comparison of the general conditions of the two groups [¯x ± s/M (P25, P75), n=28].

Index	Group A	Group B	*Z/t*	*P*
Age (years)	49.00 (48.00, 53.00)	47.00 (41.00,51.00)	-1.962	0.091
Height (cm)	159.36 ± 4.04	159.46 ± 5.45	-0.084	0.948
Weight (kg)	59.32 ± 6.80	60.64 ± 9.37	-0.064	0.548
Operation duration (min)	107.00 (74.00, 135.00)	116.50 (81.25, 160.50)	-0.647	0.517

### 3.2 Comparison of drug dosage and analgesic pump compression times

**Figure 2:**
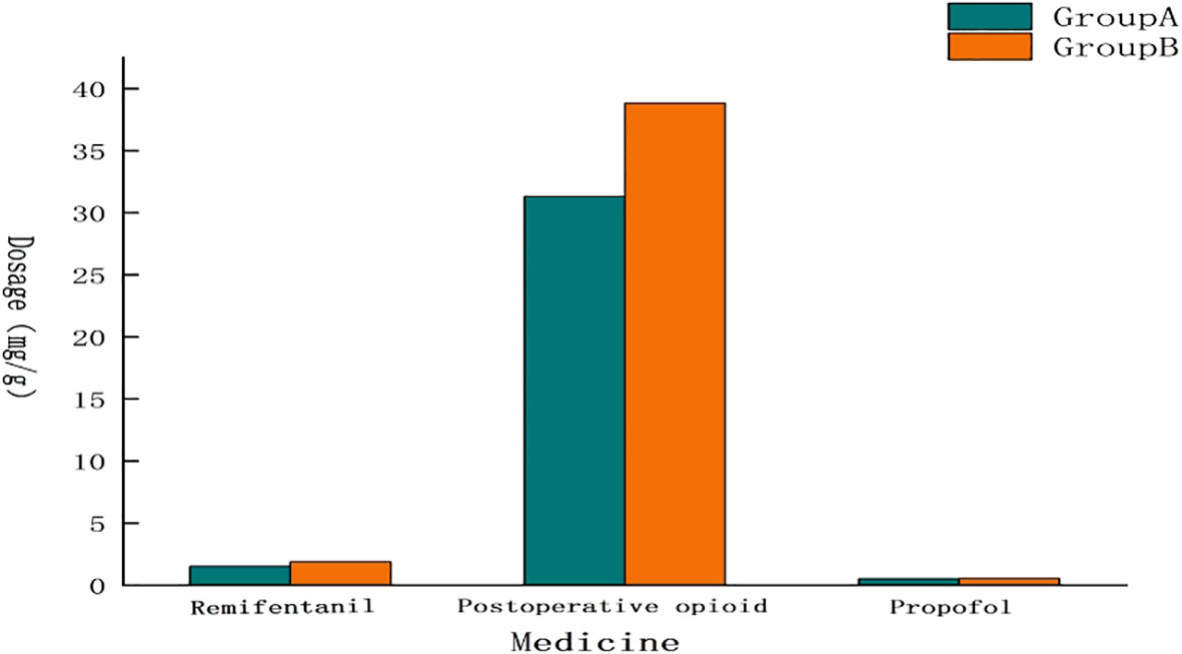
Comparison of drug dosage. The dosage of opiates and remifentanil in Group A was lower than that in Group B, and there was almost no difference in the dosage of propofol.

Compared with Group B, the dosage of remifentanil(1.49 ± 0.50 mg vs 1.86 ± 0.77 mg, P=0.039) and postoperative opioids(median, 31.79 μg with IQR 23.04-42.75μg vs median, 42.30μg with IQR 43.26-44.64μg) in Group A was reduced, and the number of analgesic pump compressions in Group A was reduced within 48 h after surgery compared with Group B(median,0.00 with IQR 1.00-2.00) vs median,1.50 with IQR 0.25-4.00, P=0.044). There was no statistical difference in the dosage of propofol between the two Groups (502.21 ± 232.08 mg vs 528.13 ± 216.18 mg, P=0.667), as shown in **Table 2** and **Figure 2**.

**Table 2:** Comparison of dosages of remifentanil, propofol, opioids, and the number of analgesic pump compressions in 48 h between the two groups [¯x ± s/M (P25, P75), n=28].

Index	Group A	Group B	Z/t	*P*
Remifentanil dosage(mg)	1.49 ± 0.50	1.86 ± 0.77	-2.115	0.039
Postoperative opioid dosage	31.79	42.30	-2.320	0.020
(μg)	(23.04, 42.75)	(43.26, 44.64)		
Propofol dosage(mg)	502.21 ± 232.08	528.13 ± 216.18	-0.432	0.667
Number of analgesic pump compressions within 48 h	0.00(1.00, 2.00)	1.50(0.25, 4.00)	–	0.044

### 3.3 VAS scores

**Figure 3:**
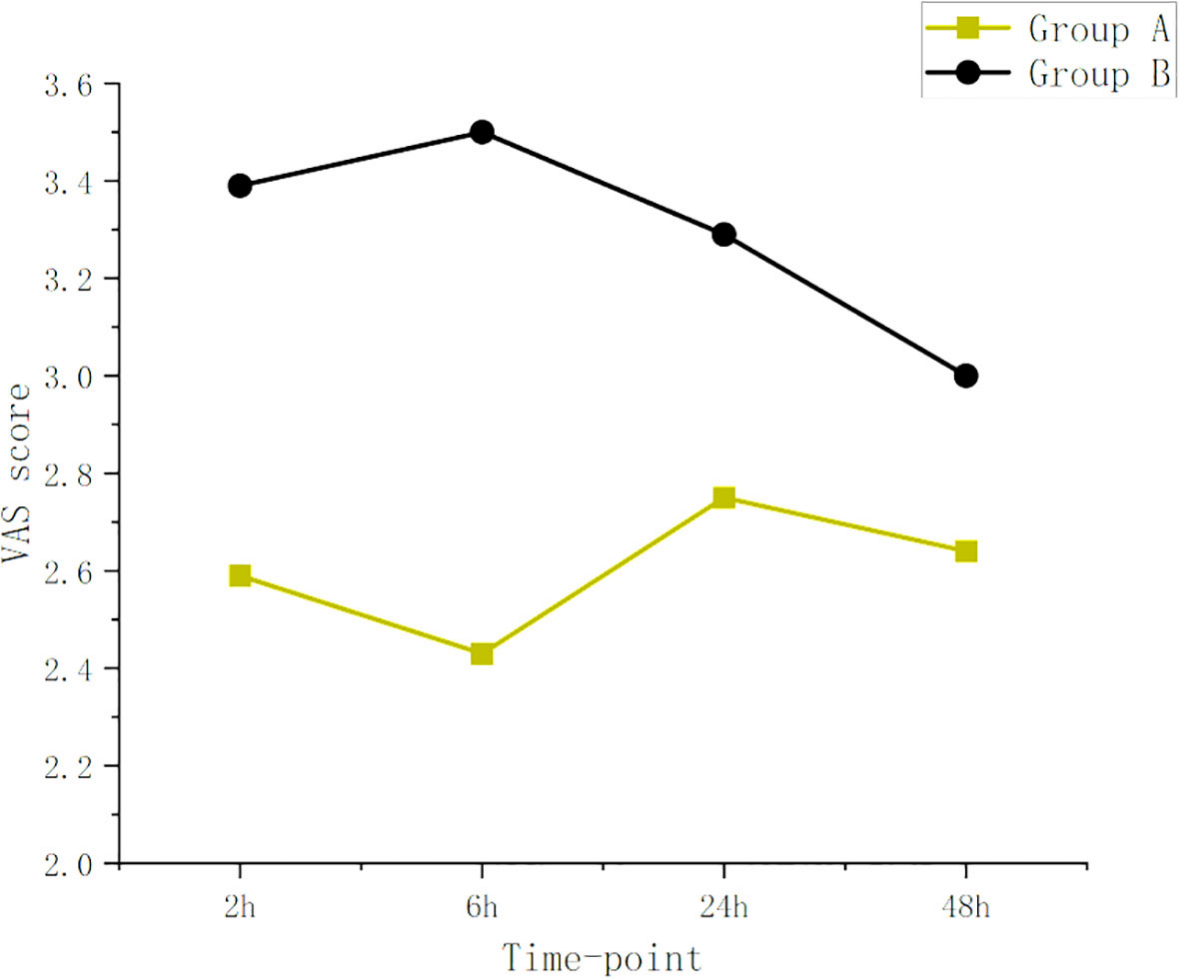
VAS dynamic. The VAS dynamic of Group A was lower than those of Group B at all time points.

**Figure 4:**
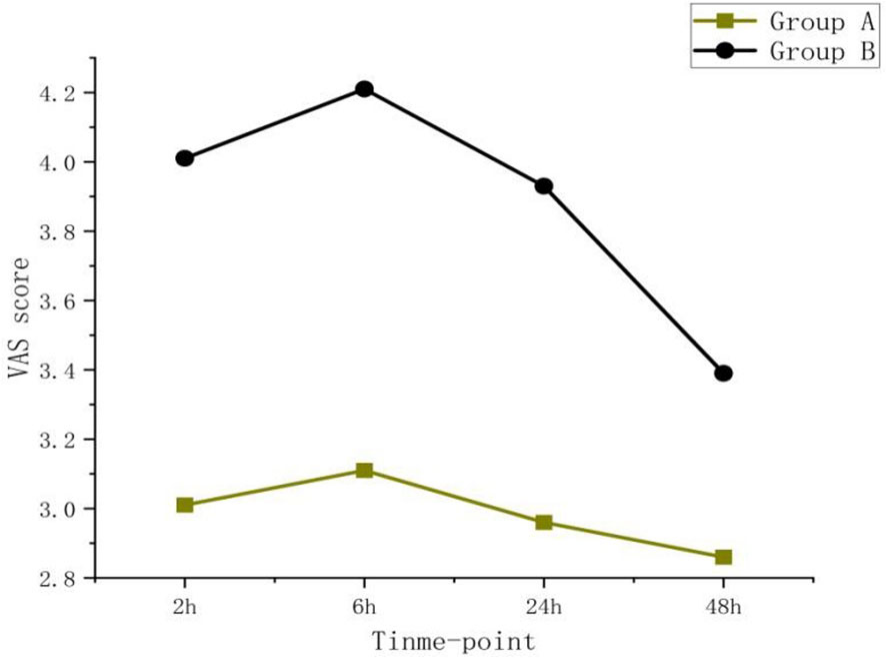
VAS static. The VAS static of Group A was lower than those of Group B at all time points, and the pain scores of both groups showed a downward trend over time.

The dynamic and static VAS scores at 2 hours (median 3.00 with IQR 2.00-3.00 vs median 4.00 with IQR 4.00-4.50, median 3.00 with IQR 2.00-3.00 vs median 3.00 with IQR 3.00-4.00, P<0.001),–6 hours (median 3.00 with IQR 2.00-3.50 vs median 4.00 with IQR 4.00-4.50, median 2.00 with IQR 2.00-3.00 vs median 3.50 with IQR 3.00-4.00, P <0.001) and 24 hours (median 3.00 with IQR 3.00-4.00 vs median 4.00 with IQR 3.50, 4.50, median 3.00 with IQR2.00-3.00 vs median 3.00 with IQR 3.00-4.00, P < 0.05) in Group A were significantly lower than those in Group B. There was no significant difference at dynamic and static VAS scores between the two groups at 48 hours(P=0.568,p=0.192). as shown in **Table 3**, **Figures 3** and **4**.

**Table 3:** Comparison of VAS scores between the two groups [M (P25, P75), n=28].

Index	Group	2h	6h	24h	48h
VAS dynamic	Group A	3.00(2.00, 3.00)	3.00(2.00, 3.50)	3.00(3.00, 4.00)	3.00(3.00, 4.00)
	Group B	4.00(4.00, 4.50)	4.00(4.00, 4.50)	4.00(3.50, 4.50)	3.00(3.00, 4.00)
	Z	-4.051	-4.424	-3.043	-0.570
	P	<0.001	<0.001	0.002	0.568
VAS static	Group A	3.00(2.00, 3.00)	2.00(2.00, 3.00)	3.00(2.00, 3.00)	3.00(2.00, 3.00)
	Group B	3.00(3.00, 4.00)	3.50(3.00, 4.00)	3.00(3.00, 4.00)	3.00(2.00, 4.00)
	Z	-3.663	-3.734	-2.517	-1.605
	P	<0.001	<0.001	0.012	0.109

### 3.4 Comparison of postoperative recovery and adverse reactions

Compared to group B, the first feeding time (median 25.00 h with IQR 20.00-30.00 h vs median 33.25 h with IQR 21.50-38.00 h,P=0.033)and anal exhaust time (median 24.00 h with IQR 21.00- 30.00 h vs median 32.00 h with IQR 24.50-38.00, P=0.045) in group A were significantly shorter, and the incidence of nausea or vomiting (4% vs 17%, P<0.001), dizziness (10%vs 21%, P=0.003)and skin pruritus (0% vs 9%, P=0.004) in group A was reduced, as shown in **Table 4**.

**Table 4:** Comparison of postoperative recovery and incidence of adverse reactions between the two groups [M(P25,P75)/n (%), n=28].

Index	Group A	Group B	Z/χ2	P
Time of first feeding (h)	25.00 (20.00, 30.00)	33.25 (21.50,38.00)	-2.127	0.033
Time of anal exhaust time (h)	24.00 (21.00, 30.00)	32.00 (24.50, 38.00)	-2.005	0.045
Nausea/Vomi ting	4 (14.30)	17 (60.70)	12.876	<0.001
Giddy	10 (32.30)	21 (67.70)	8.743	0.003
Pruritus	0 (0)	9 (32. 10)	–	0.002

## 4 Discussion

Currently, the regional nerve block technique constitutes an essential component of the multimodal analgesia strategy. The quadratus lumborum block (QLB)is widely utilized for analgesia in abdominal surgeries. It was first introduced in 2007 and has been recognized for its ability to block the lumbar plexus, its branches, trunk nerves, and sympathetic nerves (9), Furthermore, it effectively manages both somatic and visceral pain (10). A variety of distinct approaches have been developed, and numerous studies have demonstrated that the anterior approach to the quadratus lumborum block provides superior analgesic effects compared to other techniques in abdominal and lower limb surgeries (11–13), and can decrease the requirement for general anesthesia during surgical procedures. Recently, this approach has been modified within the coastal margin. Relevant studies have validated the feasibility and analgesic efficacy of this improved method (14, 15). However, this approach is associated with certain limitations, including a low block success rate, operational inconvenience, and potential drug loss due to the injection site being distant from the spine. Consequently, we implemented specific improvements in this study. In this study, the dosage of remifentanil and postoperative opioids in the modified subcostal anterior.

In this study, patients undergoing gynecologic endoscopic surgery were selected as the subjects. Gynecologic laparoscopic surgery has gained widespread application due to its numerous advantages over open surgery. Specifically, it is a minimally invasive procedure, which distinguishes it significantly from traditional open surgery. However, achieving adequate pain control following laparoscopic surgery remains a significant challenge (16); and often necessitates the use of potent analgesics, including opioids. Meanwhile, female patients undergoing laparoscopic surgery are at a significantly higher risk of experiencing postoperative nausea and vomiting (PONV) (17). Currently, with the increasing prevalence of novel opioids, the utilization of opioid analgesics has become increasingly widespread. However, their use is associated with numerous adverse effects, including respiratory depression, gastrointestinal paralysis, as well as interference with inflammatory and immune regulation (18). Choi et al. (19) conducted a prospective randomized controlled study involving 75 cases of gynecological laparoscopic surgery and demonstrated that hypoopioidization could effectively decrease the incidence of nausea and chills 30 minutes post-surgery, as well as reduce the level of hormonal stress response. These findings further substantiate the influence of opioids on patients’ postoperative recovery. Therefore, there is a critical need for the development of innovative pain management programs aimed at minimizing opioid utilization.

In our study, the modified subcostal and anterior quadratus lumborum block was found to be more effective in reducing the dosage of opioids required by patients undergoing gynecologic endoscopic surgery compared to the traditional anterior quadratus lumborum block. In addition, we conducted a 48-hour follow-up of the patients included in this study. We observed that the analgesic score within the first 24 hours postoperatively was lower in Group A compared to Group B. This difference may be attributed to the location of local anesthetic injection in the improved subcostal and anterior quadratus lumborum block technique, which facilitates the spread of the anesthetic along the thoracolumbar fascia between muscle layers, thereby blocking relevant nerve plexuses and producing an analgesic effect. No significant difference was noted in the analgesic scores at 48 hours between the two groups, likely due to the gradual metabolism of the local anesthetic over time, resulting in the absence of a nerve-blocking effect during this period. Furthermore, as time progressed, the patients’ wounds gradually healed, leading to a reduction in pain intensity. Patients in Group A required fewer instances of analgesic pump activation within 48 hours, suggesting that the modified approach not only enhances the efficacy of pain management but also diminishes the need for analgesic medication. This finding is consistent with the research results reported by Zhu Xinyan et al. (20), and may be attributed to the higher block puncture site and broader block plane associated with the improved technique targeting the anterior quadrate muscle near the inferior costal margin. Secondly, the findings of this study indicate that patients in Group A experienced fewer postoperative adverse reactions, potentially attributable to the more effective reduction of opioids achieved through the blocking method employed in Group A. Compared with Group B, the postoperative time to exhaust and the time to resume feeding were significantly reduced in Group A. This may be attributed to the fact that opioids can disrupt the normal rhythmic contractions of the gastrointestinal tract as well as mucosal secretion (21). In this study, the decreased dosage of opioids in Group A theoretically facilitated the recovery of postoperative intestinal function and minimized the impact on the central nervous system, findings which are consistent with those reported by Li Province et al. (22), these results suggest that the improved subcostal transversus abdominis plane block may more effectively promote postoperative recovery and enhance patient comfort during medical treatment. There was no difference in the propofol dosage between the two groups, which contrasts with the findings reported by Yudong et al. (23), who observed a reduction in propofol dosage via the anterior quadratus lumborum block (QLB). This discrepancy may be attributed to propofol inducing sedation through its binding to specific receptors in the central nervous system, whereas the anterior QLB exerts its effects by blocking nerve roots via drug diffusion within the thoracolumbar fascia, with no significant impact observed on the central nervous system.

In this study, the traditional anterior quadratus lumborum block (QLB) covered an extensive area from T8 to L4, while the modified anterior subcostal QLB extended from T5 to L2. Both techniques adequately fulfilled the analgesic requirements without complications such as bleeding or inadvertent entry of local anesthetics into the abdominal cavity. However, both groups experienced lower limb.muscle weakness, with Group A showing an incidence of approximately 7% (2 patients), and Group B exhibiting a higher incidence of 35.7% (10 patients). This discrepancy may be attributed to the reduced diffusion of medication to the contralateral lumbar nerve roots in the modified subcostal anterior QLB compared to the traditional QLB3 technique.

In conclusion, this study demonstrated that the modified subcostal and anterior quadrat block group required lower dosages of remifentanil and postoperative opioids compared to the traditional anterior quadrat block group. Furthermore, no significant difference was observed in propofol dosage between the two groups. The analgesia scores at 2, 6, and 24 hours postoperatively were significantly lower in Group A than in Group B. Additionally, the improved subcostal anterior quadrate block exhibited fewer adverse effects and facilitated faster gastrointestinal recovery.

Limitations: In this study, both Group A and Group B were subjected to bilateral block using the block method, with a total dose of 200 mg of ropivacaine administered. While no instances of local anesthetic toxicity were observed during the perioperative period, caution is still warranted when treating elderly patients, those who are overweight, and individuals with liver or kidney dysfunction. Because the study excluded elderly individuals, overweight subjects, and patients with liver or kidney insufficiency, the safe dosage range of ropivacaine in these populations remains to be further investigated. Secondly, the surgical operators involved in this study were not from a homogeneous group, which might have introduced a degree of variability into the experimental results. In addition, owing to the constraints of funding and experimental time, this study performed a comparative analysis solely with the traditional anterior quadratus lumborum block, without including comparisons with the conventional subcostal anterior quadratus lumborum block or other nerve blocks (e.g., Transversus Abdominis Plane Block, TABP). Such evaluations will be addressed insubsequent studies. At the same time, although the sample size of this study was calculated according to the previous pre-experiment, there were only 28 patients in each group, which was too small, which may lead to the chance of the results. Therefore, if we want to further confirm the feasibility and effectiveness of the improved method in this study, it is necessary to further expand the sample size for research.

## Data Availability

The original contributions presented in the study are included in the article/supplementary material. Further inquiries can be directed to the corresponding author/s.
